# Acidification and *γ*-aminobutyric acid independently alter kairomone-induced behaviour

**DOI:** 10.1098/rsos.160311

**Published:** 2016-09-21

**Authors:** Corie L. Charpentier, Jonathan H. Cohen

**Affiliations:** School of Marine Science and Policy, College of Earth, Ocean and Environment, University of Delaware, 700 Pilottown Road, Lewes, DE 19958, USA

**Keywords:** zooplankton, kairomones, *γ*-aminobutyric acid, pH

## Abstract

Exposure to high *p*CO_2_ or low pH alters sensation and behaviour in many marine animals. We show that crab larvae lose their ability to detect and/or process predator kairomones after exposure to low pH over a time scale relevant to diel pH cycles in coastal environments. Previous work suggests that acidification affects sensation and behaviour through altered neural function, specifically the action of *γ*-aminobutyric acid (GABA), because a GABA antagonist, gabazine, restores the original behaviour. Here, however, gabazine resulted in a loss of kairomone detection/processing, regardless of pH. Our results also suggest that GABAergic signalling is necessary for kairomone identification in these larvae. Hence, the mechanism for the observed pH effect varies from the original GABA hypothesis. Furthermore, we suggest that this pH effect is adaptive under diel-cycling pH.

## 1. Introduction

Anthropogenic CO_2_ emissions to the atmosphere are predicted to lower ocean pH by 0.14–0.43 before 2100 [1], as a result of elevated oceanic CO_2_ and subsequent changes to the marine carbonate system. Changes of this magnitude can alter sensation and behaviour in marine animals. In fact, there has been an outpouring of literature on this topic over the last several years [2]. To name a few, these elevated CO_2_ conditions can inhibit or alter olfaction [3–6], vision [7], auditory function [8], lateralization [9,10], anti-predator behaviour [11,12], visual risk assessment [13], foraging behaviour [14–16], shell assessment/homing or settlement ability [17–19] and learning [20] in marine fish, crustaceans and molluscs from both tropical and temperate environments. Such changes may have profound ecological consequences [21]. For example, larval reef fish were either attracted to or unable to detect predator odours after exposure to pH levels expected before 2100 [4]. Furthermore, mortality due to predation was higher in fish exposed to acidified seawater [6].

Nilsson *et al*. [22] proposed a neural mechanism to describe pH/CO_2_-induced behavioural disruption. Briefly, γ-aminobutyric acid (GABA) is a ubiquitous, inhibitory neurotransmitter. When GABA binds to its ionotropic GABA_A_ receptor, the receptor opens, and anions (namely chloride, Cl^−^) enter the neuron, resulting in hyperpolarization. Following decreases in environmental pH, many marine fish and crustaceans avoid acidosis by accumulating extracellular bicarbonate (HCO_3_^−^) while depleting/exchanging Cl^−^ to the external environment [23,24]. Decreases in extracellular Cl^−^ may alter the electrochemical gradient, whereby Cl^−^ exits the neuron through GABA_A_ receptors, causing depolarization. In support of this hypothesis, exposure to gabazine, a GABA_A_ antagonist, eliminates acidification effects on behaviour in both vertebrates and invertebrates, e.g. [22,25], though gabazine did not reverse increases in anxiety after exposure to acidified seawater in a juvenile rockfish [26].

Such changes to animal behaviour are often studied in the context of future predictions for ocean acidification [2]. However, present-day diel and tidal fluctuations in pH are common in shallow coastal environments, and are often greater than near-future predictions for global declines in pH [27]. Many species of crustacean zooplankton inhabit environments with diel-cycling pH and exhibit behaviours that require integration of multiple sensory modalities. For example, crab larvae maintain depth near an isolume of some lower threshold in light intensity, descending with increases in downwelling light and ascending in darkness due to negative geotaxis [28]. In addition, predator chemical cues or kairomones alter photobehaviours that are important to depth regulation [29]. For instance, kairomones from fish decrease the light intensity needed to elicit descent responses in larvae of the Asian shore crab, *Hemigrapsus sanguineus*. This leads to deeper daytime distributions, and in turn avoidance of predatory fish [30].

Because *H. sanguineus* experience diel fluctuations in pH, these larvae provide an excellent model for assessing the effect of present-day pH changes on behaviours with multisensory integration. With larval *H. sanguineus*, we assessed (i) if decreases in pH (i.e. increases in *p*CO_2_) alter the kairomone effect to photobehaviour over a diel/tidal time scale using a behavioural assay with two pH treatments, (ii) whether GABAergic signalling plays a role in the kairomone effect with a second assay assessing the dose–response relationship of gabazine to photobehaviour, and (iii) if our results support Nilsson *et al*.'s GABA hypothesis with the above behavioural assays and a comparison of extracellular Cl^−^ concentrations.

## 2. Material and methods

### 2.1. Animal collection and rearing

In September of 2014 and 2015, ovigerous female *H. sanguineus* (De Haan, 1853) were collected from Roosevelt Inlet, DE, USA. Water temperature ranged from 18 to 23°C during this time. We kept these females in the laboratory under a 14 L : 10 D cycle at approximately 22°C in seawater at a salinity of 32 practical salinity units (psu). We used either artificial seawater or local, filtered seawater (described below), and all animals were reared at ambient pH (approx. 8.1). On the day of hatching, we moved larvae into fresh seawater and water changes were conducted three times per week. Larvae were fed newly hatched brine shrimp (*Artemia franciscana*) nauplii, ad libitum.

### 2.2. Behavioural experiments

Behavioural experiments were conducted on third stage *H. sanguineus* larvae in an apparatus that mimics the underwater angular light distribution, using the same set-up, procedure, and analysis described in [30]. Each experimental group (treatment × replicate) contained approximately 15 larvae. Animals were dark-acclimated within their respective treatments for 1–3 h. We then observed and recorded their responses to 3 s downwelling light stimuli across an intensity range (10^11^–10^13^ photons m^−2^ s^−1^). All experiments were conducted at the same temperature in which animals were reared (22°C). To compare differences in photosensitivity, we determined ‘behavioural thresholds’ for each treatment. These thresholds were defined as the lowest light intensity to elicit a descent response, where the percentage of animals descending (nadir ± 60°) increased after light stimulation. A lower behavioural threshold indicates greater photosensitivity.

To determine behavioural thresholds, we first calculated the percentage descending for each group (replicate × treatment) before (dark) and after each light stimulus. Within each treatment, we compared this percentage before and after each light stimulus with one-way repeated measures (RM) ANOVAs. Given significant differences in the RM ANOVA (*p* < 0.05), Holm–Šidák *post hoc* tests were used to determine which specific light stimuli evoked a significant descent response, compared with swimming behaviour in the dark (*p* < 0.05; *p*-values corrected for multiple comparisons). The light stimulus of the lowest intensity to induce a response was deemed the ‘behavioural threshold’. Because two-way ANOVAs within treatments suggested that the percentage of animals descending did not vary between replicates or across light intensities, the mean percentage descending before each light stimulus was used as ‘percentage descending in the dark’ in the RM ANOVA analyses. All behavioural analyses were conducted in SigmaPlot 12.0 (Systat Software, San Jose, CA, USA).

#### 2.2.1. The pH experiment

Using behavioural thresholds, we assessed whether pH affects kairomone-induced photosensitivity and if the mechanism for these effects supports the proposed GABA hypothesis [22]. Prior to the behavioural assay, larvae were held in 4 l glass jars of ambient (8.1) or low (7.6) pH seawater overnight (12 h). This exposure represents the time scale over which diel/tidal pH cycles occur [27]. These containers were sealed to reduce exchange of gas or dust particles with the surrounding environment. Temperature and salinity did not change during this exposure time. Though oxygen was not measured, potential changes to dissolved oxygen would have been minimal (see details in the electronic supplementary material).

**Figure 1.**
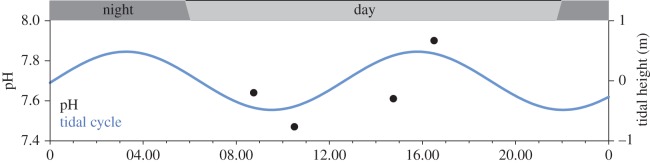
Tidal and diel pH cycles. Time series of pH (black), tidal height (m above mean lower low water, blue), and day (light grey) versus night (dark grey), measured on 9 July 2015 in Roosevelt Inlet, DE, USA, adjacent to collection site, with a sonde (6600-V2; YSI Incorporated, Yellow Springs, OH, USA).

To choose an appropriate pH range, we measured pH at the site of *H. sanguineus* collection, Roosevelt Inlet, Lewes, DE and reviewed open access pH data from a relevant offshore environment. Furthermore, on 9 July 2015, we measured pH in Roosevelt Inlet, Lewes, DE at four time points throughout the day (6600-V2 sonde with 6589 Fast-Response pH sensor; YSI Incorporated, Yellow Springs, OH, USA) (figure 1). This potentiometric electrode measures pH on the NBS scale. We recognize that this method is not ideal for measurement of seawater pH [31,32]. However, when comparing pH values during our behavioural experiment, the per cent error between pH measured on the NBS scale and the total pH scale (pH_T_; described in further detail below) was less than 2%. Our ambient treatment reflects daytime pH values found on the continental shelf of the mid-Atlantic (7.9–8.1) [33], where *H. sanguineus* spend much of their larval development [34]. We chose a low pH of 7.6 to exemplify the minimum pH these animals may experience during embryonic development, transport and diel changes in pH in this coastal environment at present day (figure 1) [33]. However, *H. sanguineus* may be exposed to pH conditions below the current minimum under future ocean acidification [1]. Seawater was regulated to pH 7.6 in a pH/CO_2_ stat system, described below.

We randomly selected 10 individuals from each pH treatment and found no difference in heart rate before and after the 12 h pH exposure (*t*_7_ = 0.8, *p* = 0.4; *t*-test), suggesting that there were no apparent differences in physiological condition between pH treatments prior to behavioural assays. After 12 h of exposure, larvae from both pH treatments were additionally exposed to the control, kairomones, gabazine (10 µM; SR-95531; Sigma-Aldrich, St Louis, MO, USA) and kairomones with gabazine for 1–3 h before we conducted assays to determine behavioural thresholds for each treatment. This pH experiment was replicated five times. Throughout the experiment, larvae were maintained in their respective pH treatments, where the ‘control’ signifies no addition of kairomones or gabazine.

Kairomone solutions were made with fish mucus, isolated from *Fundulus heteroclitus* as previously described [35] and diluted to 0.1 g mucus l^−1^ seawater. Preliminary experiments indicated that 10 μM gabazine had no effect on heart rate or survival (*t*_8_ = 0.1, *p* = 0.9; *t*-test), suggesting that gabazine at this concentration was not toxic to these crab larvae. Furthermore, we chose to use gabazine to inhibit GABAergic signalling in this study, because (i) gabazine has effectively reversed the effects of acidification in both vertebrates and invertebrates, e.g. [22,25] and (ii) gabazine functions as a competitive antagonist in other arthropods, e.g. fruit fly, *Drosophila* [36] and locust, *Locusta migratoria* [37] and in an aquatic invertebrate, *Hydra vulgaris* [38,39].

#### 2.2.2. The gabazine dose–response experiment

We conducted a kairomone/gabazine dose–response experiment to better determine the role of GABAergic signalling in the kairomone effect to photobehaviour. Larvae in ambient pH were exposed to the control, kairomones, gabazine only (10 µM) or kairomones with gabazine at three concentrations (10, 1 and 0.1 µM) for 1–3 h. As before, we determined behavioural thresholds for each treatment, and this dose–response experiment was replicated five times.

### 2.3. Analysis of extracellular fluid

Following the pH experiment, individuals (*n* ∼ 30) from ambient and low pH treatments were blotted dry and homogenized with a micropestle. We extracted extracellular fluid (ECF) with a syringe. These samples were diluted 1 : 5000 with eluent buffer (3.2 mM sodium carbonate, 1.0 mM sodium bicarbonate, 6.5% v/v acetone), filtered (0.2 µm) and stored at 4°C for subsequent analysis of ECF Cl^−^ by anion chromatography (Metrohm AG, Herisau, Switzerland). We compared Cl^−^ concentrations between animals exposed to ambient and low pH with a *t*-test, and results were deemed significant if *p* < 0.05. This analysis was conducted in R (R Core Team, 2015, Vienna, Austria).

To better understand the pH effect on electrochemical gradients, we also conducted a separate experiment with paired measurements of ECF Cl^−^ and osmolality. At least 1 day post-hatching, stage 1 *H. sanguineus* larvae were placed in artificial seawater with a salinity of 15, 30 or 40 psu at ambient pH or a salinity of 30 psu at low pH. After 24 h of exposure, ECF was exacted from groups of approximately 40 individuals in each treatment. After extraction, we measured ECF osmolality with a vapour pressure osmometer (Vapro 5600, ELITech Group, Puteaux, France) and chloride (Cl^−^) with ion chromatography, as described above. This paired experiment was repeated twice. Samples were diluted prior to analysis, and we corrected for this dilution. Perhaps due to our method of extraction, values for both osmolality and Cl^−^ appeared lower than expected [40]. Hence, we also multiplied all values by a common factor (1.99) to reach expected values, nearly isosmotic to seawater [40]. These corrections had no effect on the outcome of our statistical comparisons (see details in the electronic supplementary material), and all samples were extracted, measured and analysed in the same fashion. Here, we report corrected values of osmolality and Cl^−^ for both the pH and paired experiment. Contrary to the behavioural experiments, we used first-stage zoeae in this chloride-osmolality analysis to increase the ECF sample volume, as some broods experience high mortality over larval development (e.g. due to maternal effects) [41]. Because zoeae are osmoconformers at both beginning and intermediate stages of development [40], the use of younger zoeae should not change our interpretation.

### 2.4. Seawater chemistry

In all experiments, we controlled for the presence of kairomones. Furthermore, in the pH experiment, seawater for rearing and both pH treatments was artificial seawater (Instant Ocean, Spectrum Brands, Blacksburg, VA, USA). In the gabazine dose–response experiment, however, seawater for rearing and experiments was collected from Indian River Inlet, Delaware, USA. Biologically active molecules were removed by ultrafiltration at 100 kDa (GE Life Sciences UFP-100-C-5A) and ageing in darkness for more than a week [35]. We chose to use artificial seawater for the pH experiment because we regulated pH in a laboratory set-up that operates with artificial seawater exclusively.

In the pH experiment, the low pH treatment was regulated to pH 7.6 with a pH/CO_2_ stat system, which consisted of a custom program in LabVIEW (National Instruments, Austin, TX, USA) that communicated to a relay board to activate flow of CO_2_ and CO_2_-scrubbed air, given feedback from a differential pH electrode (Hach Company, Loveland, CO, USA). Air bubbled directly into a sump, and water was retrieved from this sump for experiments. Owing to spatial and temporal constraints, the ambient pH was not regulated with the described pH/CO_2_ stat system. However, we used the same salinity and artificial seawater mix in both to obtain similar carbonate parameters. Before and after pH exposure, we made pH measurements with a combination electrode (Thermo Fisher Scientific, Inc., Beverly, MA, USA) using the NBS scale. Within the sealed containers, we found that pH increased slightly during exposure time in the low pH treatment (approx. 0.1), but this increase was consistent across replicates. As stated previously, we understand that pH measurements with potentiometric electrodes on the NBS scale are not ideal for measuring seawater pH [31,32]. Hence, we report our ambient and low pH treatments on the total pH scale (pH_T_), calculated from measured values of dissolved inorganic carbon (DIC) and total alkalinity (TA), described below.

At the time of exposure, seawater samples were fixed with saturated mercury bichloride [42,43] and stored at 4°C for future analysis. TA was analysed by Gran titration [44], using the open-cell method with a semi-automatic titration system (Apollo Scitech, LLC, Newark, DE, USA) [45,46]. To determine the concentration of DIC, samples were acidified with phosphoric acid, and the quantity of extracted CO_2_ gas was then measured with an infrared gas analyser (Apollo Scitech, LLC). Both TA and DIC measurements were taken at a precision level of about 2 µmol kg^−1^ [46] and calibrated against a certified reference material (provided by A.G. Dickson, Scripps Institution of Oceanography). Values for *p*CO_2_ (μatm) and pH_T_ were calculated with the Excel MACRO CO2Sys developed by Pierrot *et al*. [47] with [*B*]*_T_* from [48], *k*1, *k*2 from [49,50] and *K*s from [51] (table 1). Though values of TA are higher than would be expected at a salinity of 32 in the ambient pH treatment (table 1), our pH calculations and results should not be affected by this difference (details in the electronic supplementary material).

**Table 1.** Parameters of the carbonate system during experiments. Values are means ± s.e. Dissolved inorganic carbon and total alkalinity are represented by DIC and TA, respectively. Calculation of *P*_CO_2__ and pH_T_ was made using the Excel MACRO CO2Sys developed by Pierrot *et al.* [45], with [*B*]*_T_* from [46], *k*1, *k*2 from [47,48], and *K*s from [49]. Temperature and salinity were 22°C and 32°C, respectively, in both pH treatments.

	ambient	low
pH_T_	8.1 ± 0.06	7.6 ± 0.01
*p*CO_2_ (μatm)	461 ± 97	1380 ± 30
TA (μmol kg^−1^)	3021 ± 85	2546 ± 17
DIC (μmol kg^−1^)	2612 ± 86	2448 ± 13

## 3. Results and discussion

**Figure 2.**
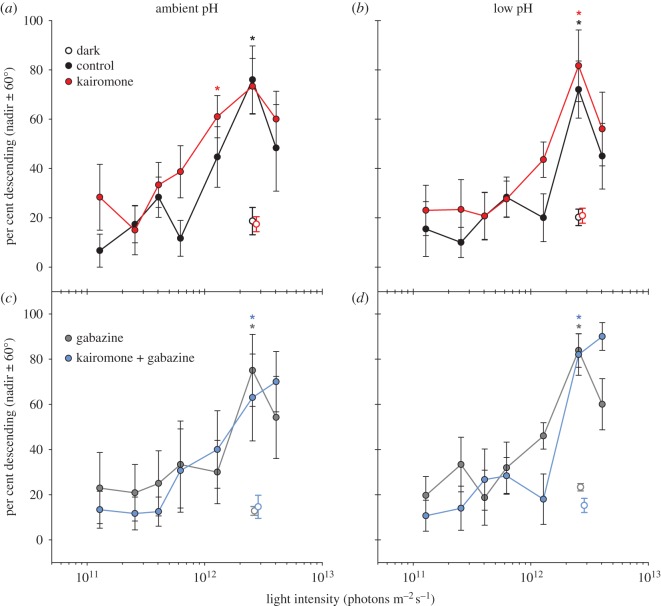
Effect of pH on kairomone-induced photosensitivity. Mean ± s.e. per cent descending of larval *Hemigrapsus sanguineus* in darkness (unfilled) and after exposure to downwelling light (filled). Before experiments, animals were exposed to (*a*,*c*) ambient pH or (*b*,*d*) low pH and to control (black), gabazine only (10 µM, grey), kairomones (red) or kairomones with gabazine (blue). Behavioural thresholds, or light intensity that elicits a descent response, are indicated with asterisks and were determined by one-way RM ANOVAs with Holm–Šidák *post hoc* tests (*p* < 0.05; *n* = 5 replicates with approximately 15 larvae per replicate, except *n* = 4 replicates in ambient, gabazine-only treatment).

We assessed the effect of pH and the role of GABAergic signalling in kairomone-induced changes to photobehaviour by comparing the behavioural threshold, i.e. the lowest light intensity to elicit a descent response, between pH and kairomone/gabazine treatments. For all treatments in the pH experiment, percentage descending was greater after light stimulation than in darkness (*F*_6,7_ = 2.6–11.2, *p* < 0.04; one-way RM ANOVA). Under ambient pH conditions (approx. 8.1), the behavioural threshold of kairomone-exposed larvae was 1.28 × 10^12^ photons m^−2^ s^−1^ (*p* = 0.03; Holm–Šidák *post hoc* test), nearly two times less than those not exposed to kairomones (2.55 × 10^12^ photons m^−2^ s^−1^; *p* = 0.006; figure 2*a*). All those in low pH had the same threshold as the ambient control (*p* < 0.01; figure 2*b*). Hence, exposure to acidified seawater removed kairomone-induced photosensitivity in *H. sanguineus*. Past studies attribute similar changes in behaviour and sensation to increases in CO_2_, reviewed in [2]. Given differences in carbonate chemistry between our pH treatments (table 1), we cannot isolate which particular carbonate parameter(s) may be driving these changes. Furthermore, addition of gabazine with and without kairomones also resulted in the same threshold as the control, regardless of pH (*p* < 0.04; figure 2*c,d*). Previous work suggests that such changes to behaviour are due to disrupted function of GABAergic signalling, because gabazine restored the loss of behavioural function in both vertebrates and invertebrates, e.g. [22,25]. Here, however, inhibiting GABA_A_ receptors at low pH did not restore the ‘normal’ kairomone effect, suggesting that the observed pH effect is mediated by a different mechanism. Hamilton *et al*. [26] similarly found that gabazine did not reverse increases in anxiety after exposure to acidified seawater in a juvenile fish. However, a GABA agonist, muscimol, added to increases in anxiety at low pH. Because muscimol is known to decrease anxiety [52], changes to the ‘normal’ function of this agonist ultimately support Nilsson *et al*.'s hypothesis of reversed function of GABA_A_ receptors in acidified conditions. Naturally, further work examining the effects of muscimol on behaviour would offer greater certainty in confirming or contradicting Nilsson *et al*.'s hypothesis.

To better understand the role of GABAergic signalling in kairomone-induced photosensitivity, we assessed the effect of gabazine across a concentration gradient at ambient pH in a dose–response experiment. Again, percentage descending was greater after light stimulation than in darkness (*F*_5,6_ = 8.0–17.8, *p* < 0.001, one-way RM ANOVA). Here, the behavioural threshold was lower in larvae exposed to kairomones (*p* = 0.013; Holm–Šidák *post hoc* test) and kairomones with gabazine at the lowest concentration (0.1 µM; *p* = 0.017), compared with those exposed to the control, gabazine only and kairomones with gabazine at higher concentrations (10, 1 µM; *p* < 0.02; table 2). As in our pH experiment, gabazine resulted in a loss of kairomone-induced photosensitivity. In this dose–response experiment, we also find that the effect of gabazine decreased with concentration, suggesting that GABAergic signalling is required to induce the ‘normal’ kairomone effect. Because swimming behaviour did not differ between control and gabazine treatments, GABAergic signalling probably plays a role in kairomone detection and/or processing. In the spiny lobster, *Panulirus argus*, opening of GABA_A_ receptors induced presynaptic inhibition of olfactory receptor neurons that regulate input to the central nervous system [53]. Perhaps *H. sanguineus* lose a similar regulatory step after exposure to gabazine or low pH.

**Table 2.** Effect of gabazine on kairomone-induced photosensitivity of stage 3 *Hemigrapsus sanguineus* larvae. Behavioural thresholds were determined by one-way RM ANOVAs with Holm–Šidák *post hoc* tests, comparing percentage descending before and after exposure to a range of light intensities (*n* = 5 replicates, approximately 15 larvae per replicate), conducted at ambient pH (approx. 8.1).

	behavioural threshold (photons m^−2^ s^−1^)
control	6.52 × 10^11^
kairomones	4.10 × 10^11^
gabazine (10 μM)	6.52 × 10^11^
kairomones + gababzine (10 μM)	6.52 × 10^11^
kairomones + gababzine (1 μM)	6.52 × 10^11^
kairomones + gababzine (0.1 μM)	4.10 × 10^11^

Nilsson *et al*. [22] hypothesized that altered behaviour and neural function at low pH is due to changes in the electrochemical gradient during an acid disturbance, namely accumulation of HCO_3_^−^ that is compensated by reduced extracellular Cl^−^. Contrary to this hypothesis, extracellular Cl^−^ did not vary between larvae exposed to ambient or low pH, with mean ± s.e. concentrations of 292 ± 63 and 282 ± 94 mM, respectively (*n* = 5; *t*-test, *t*_8_ = 0.08, *p* = 0.9). In an additional paired chloride–osmolality analysis, both Cl^−^ and osmolality increased with seawater salinity. Again, Cl^−^ was similar between the two pH treatments. Though our small sample size (*n* = 2) inhibited robust statistical analysis, osmolality appeared higher at low pH (table 3), suggesting an increase of other osmolytes, which could include HCO_3_^−^. In addition to Cl^−^, GABA_A_ receptors have a lesser affinity for HCO_3_^−^ [54]. Hence, it is possible that the observed pH effect is due to changes in GABAergic signalling during acid–base regulation. However, our behavioural results suggest that the exact mechanism differs from that proposed by Nilsson *et al.* [22]. Alternatively, ion regulation may vary significantly in larval crabs, relative to Nilsson *et al*.'s fish-based hypothesis. For instance, extracellular Cl^−^ increased after exposure to acidified seawater in a hermit crab [3]. Though we used gabazine concentrations far below known thresholds for toxicity in another crustacean zooplankter (*Daphnia*) [55] and we found no difference in heart rate or mortality after gabazine exposure, we cannot entirely eliminate the possibility that gabazine may be acting as an olfactory toxin in these larval crabs. In addition, we recognize the possibility that the effect or even toxicity of gabazine may have varied between seawater types in the pH and dose–response experiment (artificial and filtered seawater, respectively). However, this is unlikely, as we observed similar responses to gabazine (10 µM) at ambient pH in both.

**Table 3.** Osmolality (mOsm kg^−1^) and Cl^−^ concentration ([Cl^−^], mM) in extracellular fluid (ECF) of stage 1 *Hemigrapsus sanguineus* larvae after exposure to various salinities. Values shown are means (±s.e.; *n* = 2).

	seawater		ECF	
pH	salinity	osmolality	osmolality	[Cl^−^]
8.1	15	307 ± 38	260 ± 60	229 ± 40
8.1	30	680 ± 36	700 ± 200	292 ± 90
8.1	40	907 ± 67	1069 ± 160	304 ± 89
7.6	30	704 ± 69	1059 ± 70	289 ± 81

To date, most studies assess the effects of CO_2_ and/or pH on behaviour in the context of future global ocean acidification [2]. However, in estuarine and coastal habitats, present-day pH fluctuations are influenced by biological activity, where pH generally decreases at night and increases during the day [56]. We found a similar pattern at our *H. sanguineus* collection site (figure 1), and the observed pH effects to behaviour occurred over a time scale similar to these cycles. Because our pH measurements were taken over the course of one day with fair weather, we recognize that pH cycles may also vary over small and large spatial scales and fluctuate due to processes other than biological activity (e.g. river and groundwater input) [57]. Still, we propose that the observed pH effect on larval crab behaviour may be adaptive. Larval crabs maintain depth near an isolume defined by their threshold for light detection [28], and larvae become more photosensitive after kairomone exposure. Such behaviours contribute to diel vertical migration, a long-proposed strategy for avoidance of visual predators [29]. When pH is highest (daytime), larvae would be most sensitive to kairomones, migrating into deeper, darker water when the threat of visual predators is greatest. Kairomone-induced changes to photobehaviour are thought to result in some cost, e.g. separation from food [58]; therefore, losing the kairomone effect during times of least danger (night-time, when pH is lowest) would provide larvae with an adaptive advantage.

## 4. Conclusion

In this study, we assessed if decreases in pH alter the kairomone effect to zooplankton photobehaviour over a diel/tidal time scale and whether altered function of GABA_A_ receptors during acid–base regulation was the mechanism for this change. We found that exposure to low pH removed the kairomone effect to photobehaviour, suggesting that pH inhibits kairomone detection and/or processing over these time scales. Furthermore, GABAergic signalling probably plays a role in detection/processing of kairomones, as addition of gabazine at ambient pH also removed the kairomone effect to photobehaviour and this removal was dependent upon gabazine concentration. Unlike Nilsson *et al*.'s GABA hypothesis, however, gabazine did not restore this pH effect and extracellular Cl^−^ concentrations did not vary between the ambient and low pH treatments. Hence, our results suggest that changes in Cl^−^ transport through the GABA_A_ receptor may not be the sole mechanism for pH effects to sensation and behaviour. Furthermore, acidification and GABAergic signalling independently altered the ‘normal’ kairomone effect to photobehaviour in these zooplankton. Contrary to previous studies, which focused on the deleterious effects of pH in the context of future global ocean acidification, we suggest that the loss of this kairomone effect after exposure to low pH may actually be adaptive in zooplankton that experience present-day diel fluctuations in pH, common in coastal environments.

## Supplementary Material

Supplementary material for Acidification and GABA independently alter kairomone-induced behaviour (Document contains all supplementary information).

## Data accessibility

All supporting data not included here are made available in the electronic supplementary material and at doi:10.5061/dryad.5jn6b [59].
