# Supplementary material for: Acidification and γ-aminobutyric acid independently alter kairomone-induced behaviour
Source: R Soc Open Sci. 2016 Sep 21;3(9):160311. doi: 10.1098/rsos.160311 (PMC5043316; doi:10.1098/rsos.160311)
Supplement: Supplementary material for Acidification and GABA independently alter kairomone-induced behaviour (Document contains all supplementary information). [file rsos160311supp1.pdf]

Supplementary material

for

Acidification and GABA independently alter kairomone-induced behaviour

Corie L. Charpentier* and Jonathan H. Cohen

*Corresponding author: Email: charpecl@udel.edu

2. Methods

2.2.1. The pH experiment

Though we did not measure oxygen during the pH experiment, potential changes to dissolved oxygen (DO) in our sealed containers would have been minimal. In both pH treatments, we placed ~ 65 zoeae in 4 L of artificial seawater (Instant Ocean, Spectrum Brands, Blacksburg, VA, USA) for 12 h at 32 practical salinity units (psu) and 22 °C. During the third zoeal stage, Marsh et al. [1] found that zoeae consume approximately 6 *n*mol O_2 h^{-1} $zoea^{-1}$ at a similar temperature (21 °C). Artificial seawater at 32 psu and 22 °C has a DO concentration of ~ 7.0 mg L^{-1} (Pro2030; YSI Incorporated, Yellow Springs, OH, USA). Using the simple calculation shown below, we determined that DO should have decreased by only 0.04 mg L^{-1} during our 12 h exposure, resulting in a percent decrease of 0.5%.

$$\Delta \text{DO} = \text{O}_2 \text{ consumption } \left(\frac{\text{mol O}_2}{\text{h} \times \text{zoea}}\right) \times \text{Molar mass } \left(\frac{\text{mg O}_2}{\text{mol O}_2}\right) \times \frac{\text{\# of zoeae}}{\text{Container volume (L)}} \times \text{Exposure time (h)}$$

2.3. Analysis of extracellular fluid

We multiplied all values for osmolality and Cl^- by a common faction (1.99), because our original concentrations were lower than expected values for larval crab extracellular fluid (ECF), nearly isosmotic to seawater [2]. Here, we defend that these corrections had no effect on the outcome of our statistical comparisons. Further, we extracted ECF following the pH behavioural assay. Mean ± se ECF Cl^- concentrations were 146 ± 40 m*M* in ambient pH and 141 ± 47 m*M* in low pH before the correction. Following the correction, concentrations were 292 ± 63 m*M* in ambient pH and 282.0 ± 94 m*M* in low pH. To ensure that this correction did not alter our statistical interpretations, we conducted t-tests comparing Cl^- concentrations between pH treatments before and then after the correction. In both analyses, we found no significant difference between the two pH treatments ($t(8) = 0.08$, $P = 0.93$). This statistical analysis was conducted in R (R Core Team, 2015, Vienna, Austria).

2.4. Seawater chemistry

The total alkalinity (TA) values of our ambient pH treatment were higher than expected, given a salinity of 32 practical salinity units (psu, table 1). With few exceptions (e.g. calcium carbonate dissolution), TA is generally conservative and correlated with salinity. As mentioned in our methods, the same salinity and artificial seawater mix (Instant Ocean, Spectrum Brands, Blacksburg, VA, USA) were used in both the ambient and low pH treatments. However, the seawater for the low pH treatment was extracted from a system that had been circulating in a large sump for several weeks, while seawater for the ambient pH treatment was made on the day of use. Though Instant Ocean salt mix and deionized water were mixed until the solution was clear (i.e. no visible salt crystals), it is possible that salt continued to dissolve far after our initial

mix, which would impact salinity and therefore TA. Further, TA was analyzed several weeks after completion of behavioural experiments. Fortunately, salinity of Instant Ocean seawater did not increase significantly over a 24 h period (table S1). Hence, our behavioural and ECF Cl^- results should not have been influenced by salinity differences between the two pH treatments. To further investigate whether TA of recently mixed Instant Ocean salt mix changes over longer time periods in storage, we analyzed TA of samples at 32 psu and found that TA was 2365 and 3253 µmol kg^{-1}, 24 and 72 h after solution was made, respectively. This could explain higher TA values at ambient pH. Regardless, pH should have been maintained over this time period, because (1) we properly eliminated biologically-induced changes to carbonate chemistry (e.g. addition of saturated mercury bichloride) and (2) seawater has a high buffering capacity, where increases in TA are correlated with dissolved inorganic carbon (DIC) as HCO_3^- contributes significantly to both. As secondary evidence for this, the mean ± se pH_{NBS} measurements at the time of the experiment were similar (8.1 ± 0.02) to calculated values of pH_T shown in Table 1 (8.1 ± 0.06).

Table S1. Salinity of artificial seawater (Instant Ocean) at 0 and 24 h after dissolution of salt mixture into deionized water

Initial salinity (0 h)	Salinity after 24 h
32.4	32.4
32.3	32.7
29.1	29.1

Seawater was considered "dissolved" after mixture was clear (i.e. no apparent salt crystals), and salinity was measured with a handheld meter (Pro2030; YSI Incorporated, Yellow Springs, OH, USA). There were no significant differences in salinity between 0 and 24 h after dissolution ($t(2) = -1$, $P = 0.4$; paired t-test).

References

[1] Marsh AG, Cohen S, Epifanio CE. 2001 Larval energy metabolism and physiological variability in the Asian shore crab *Hemigrapsus sanguineus*. *Mar. Ecol. Progr. Ser.* **218**, 303–309. (doi:10.3354/meps218303)

[2] Anger K, Torres G, Charmantier-Daures M, Charmantier G. 2008 Adaptive diversity in congeneric coastal crabs: Ontogenetic patterns of osmoregulation match life-history strategies in *Armases* spp (Decapoda, Sesarmidae). *J. Exp. Mar. Biol. Ecol.* **367**, 28–36. (doi:10.1016/j.jembe.2008.08.009)
